# Authors’ Reply to: Minimizing Selection and Classification Biases Comment on “Clinical Characteristics and Prognostic Factors for Intensive Care Unit Admission of Patients With COVID-19: Retrospective Study Using Machine Learning and Natural Language Processing”

**DOI:** 10.2196/29405

**Published:** 2021-05-26

**Authors:** Jose Luis Izquierdo, Joan B Soriano

**Affiliations:** 1 Universidad de Alcalá Madrid Spain; 2 Hospital Universitario de La Princesa Madrid Spain

**Keywords:** artificial intelligence, big data, COVID-19, electronic health records, tachypnea, SARS-CoV-2, predictive model, prognosis, classification bias, critical care

We acknowledge the letter by Martos Pérez et al [1] and take this opportunity to clarify related issues from our publication [2]. Of our 10,504 patients with COVID-19, 2737 (26.5%) were tested with PCR (polymerase chain reaction). Within the 5 provinces of Castilla-La Mancha, the province that tested the most was Toledo (28.9%), while the least was Guadalajara (21.2%). Those patients in whom PCR was performed were 6.5 years older (63.0 vs 56.5 years). All these differences were highly statistically significant.

You must take into account that our study period was from March 1 to 29, 2020, and including only microbiologically confirmed cases or prolonging the period of inclusion would have resulted in a biased assessment. From March 30, 2020, onwards, most intensive care units (ICUs) at our hospitals collapsed and ICU admissions were highly distorted due to a lack of beds. As we commented in the *Discussion* section, the ICU capacity in Castilla-La Mancha during the study period had not yet been compromised, which protects against possible bias in our training data (all patients requiring critical care were indeed admitted to the ICU). Therefore, it is unlikely that the absence of a confirmed diagnosis with PCR during the first weeks of the pandemic influenced our results. This was a generalized situation throughout Spain and in most European countries early in 2020. At that time, when a patient was hospitalized, a wide battery of viruses was considered for which there were reagents before performing PCR for coronaviruses. Patients seen during the month of March, in the midst of an avalanche of COVID-19 cases in our region, with negative tests for other viruses and clinical, radiologic, and blood tests highly compatible, did not raise doubts about their diagnosis of COVID-19, and the probability of error was considered negligible [3-5]. For all these reasons, bias in our AI (artificial intelligence) algorithms is highly unlikely. We, however, agree that admission to the ICU can be related to many factors. One strength of our study is that it analyzes the usual clinical practice in the whole population cared for in an entire health care region of Spain during a period when the lack of beds was not a limiting factor. It was not a sample—it was the entire population. Finally, our study objective was not mortality. In other studies, when we addressed mortality, the study period was extended to reliably collect this variable [6,7].

## Abbreviations

AI: artifical intelligence
ICU: intensive care unit
PCR: polymerase chain reaction
